# Eosinophilic esophagitis and esophageal microbiota

**DOI:** 10.3389/fcimb.2023.1206343

**Published:** 2023-08-03

**Authors:** Xiaohan Zhang, Nana Zhang, Zikai Wang

**Affiliations:** ^1^ Microbiota Division, Department of Gastroenterology and Hepatology, The First Medical Center, Chinese PLA General Hospital, Beijing, China; ^2^ Medical School, Nankai University, Tianjin, China; ^3^ National Clinical Research Center for Geriatric Diseases, Chinese PLA General Hospital, Beijing, China

**Keywords:** eosinophilic esophagitis, esophageal microbiota, mechanisms, microbiome, treatment

## Abstract

Eosinophilic esophagitis (EoE) is an antigen-mediated chronic inflammatory disease of the esophagus, the prevalence of which has steadily increased in recent years. The pathogenesis of EoE is not yet well-defined; however, recent studies have demonstrated that the esophageal microbiota is an essential regulator of physiological and pathological processes of EoE. Currently, research on EoE and microbiota is an emerging field of study that is receiving increasing attention. Here, we review existing EoE-related esophageal microbiota studies to explore the potential mechanisms underlying esophageal microbiota-mediated EoE. The esophageal microbiome is altered in patients with EoE. Although α diversity is usually not significantly different, an increase in *Haemophilus* and a decrease in *Firmicutes* were observed in EoE patients. The role of microbiota in initiating and perpetuating inflammation is not fully understood. Current evidence suggests that the penetration of microbiota leads to the activation of epithelial cells as well as innate and adaptive immune cells, with the subsequent release of cytokines, leading to immune responses and inflammation. The involvement of toll-like receptors in EoE also supports the potential role of the microbiota in the progression of this disease. While EoE-induced inflammation can also lead to alterations in the local microbiome. Moreover, dietary modifications, proton pump inhibitors, and corticosteroids can modulate the esophageal microbiota; however, definitive conclusions about the alterations of microbes after treatment cannot be drawn. These findings provide promising avenues for future studies.

## 1 Introduction

Eosinophilic esophagitis (EoE) is a chronic inflammatory disease characterized by clinically significant esophageal dysfunctions (Choudhury and Baker, 2020), such as dysphagia, food impaction, vomiting, and abdominal pain, accompanied by atopic dermatitis, food allergy, asthma, and allergic rhinitis (Capucilli and Hill, 2019). Histopathological manifestations of EoE include intraepithelial eosinophilic infiltration (≥15/high magnification view) and esophageal epithelial remodeling, leading to esophageal stricture (Khokhar et al., 2022). The prevalence of EoE has steadily increased in recent years from 5–10/100,000 person-years to 20/100,000 person-years (Arias and Lucendo, 2020). A systematic review estimated the combined prevalence of EoE at 34.4 per 100,000 individuals, with higher prevalence in adults than in children (Navarro et al., 2019). EoE, the second most common chronic or recurrent esophageal disease after gastroesophageal reflux disease, is the leading cause of dysphagia and food impaction in children, adolescents, and young adults in Europe and North America affecting quality of life (Arias and Lucendo, 2019).

The pathogenesis of EoE is not well-defined and is currently thought to be the outcome of a confluence of genetic, environmental, and immunological factors. The esophageal epithelium of susceptible individuals with risk factors such as a history of antibiotic application early in life, genetic background, or atopic status, can release alarmin, IL-33, and thymic stromal lymphopoietin following antigenic stimulation. These cytokines stimulate the secretion of IL-13, IL-4, and IL-5 by T helper (Th2) cells, leading to the migration of eosinophils into the esophagus, disruption of esophageal mucosal barrier integrity, and eventual esophageal fibrosis in patients with EoE (Votto et al., 2020; Muir and Falk, 2021; Racca et al., 2021). Recently, the role of esophageal microbes in EoE has attracted attention. The development of 16S rRNA gene sequencing has been instrumental in investigation of the esophageal microbiota. Emerging studies suggest that patients with EoE may have a unique esophageal resident microbiome that interacts with the host to influence cellular activities and metabolic processes (Busing et al., 2021). However, whether alteration of the esophageal microbiota is a contributing factor, or the outcome of esophageal inflammation related to EoE remains controversial. Primary management of EoE includes dietary modifications, pharmacological drugs such as proton pump inhibitors (PPIs), and hormone therapy. The esophageal microbiota is altered in the pre- and post-treatment phases and may be an indicator of EoE therapeutic outcomes. This review integrates EoE-related esophageal microbiota studies conducted over the past decade to explore the potential clinical value of the esophageal microbiota as a biomarker, providing a foundation for further research and treatment of EoE.

## 2 Esophageal microbiota characteristics associated with EoE

Dysbiosis of the esophageal microbiota occurs in children and adults with EoE and is characterized by an increase in the abundance of *Haemophilus* and a decrease in that of *Firmicutes* compared to healthy controls (**Tables 1**, **2**). Benitez et al. compared the esophageal bacteria between 33 children with EoE and 35 healthy controls. They found that the esophageal mucosa of children with EoE was dominated by *Neisseria* and *Corynebacterium*, whereas that of healthy children was dominated by *Streptococcus* and *Atopobium* (Benitez et al., 2015). They also analyzed and compared the α diversity of esophageal microbiota among children with EoE and healthy controls and found nonsignificant differences (Benitez et al., 2015; Parashette et al., 2022). Another study found that untreated children with EoE had a lower abundance of *Firmicutes* and a higher abundance of *Haemophilus* than healthy controls (Harris et al., 2015), which is consistent with Benitez’s second study on the microbiome of EoE (Benitez et al., 2021). Despite differences in taxa, conclusions drawn from the α diversity of the esophageal microbiota in adults with EoE have been consistent. A study that included 16 patients found that *Firmicutes* was more abundant in patients with inactive EoE, whereas *Actinobacillus* and *Alloprevotella* were abundant in adults with active EoE (Ghisa et al., 2020). Laserna-Mendieta and colleagues compared the esophageal microbiota of 30 patients with EoE and 10 healthy controls and found that the abundance of *Porphyromonas* and *Parvimonas* was lower in patients with EoE than in healthy individuals (Laserna-Mendieta et al., 2021). Smith et al. compared the esophageal microbiota of 6 untreated children with EoE with those of 18 healthy controls; however, they did not observe any significant differences in specific microbes in children with EoE (Smith et al., 2015). Similarly, a recent study found no significant alterations in adult patients with EoE (Johnson et al., 2021).

**Table 1:** Summary of studies about the microbiota in pediatric EoE.

Study	Study population	Medication	Sample	Methods	Findings
Benitez et al. (2015)	68 pediatric participants (33 EoE, 35 non-EoE controls)	66 on PPIs	oral swabs and esophageal biopsy samples	16S rRNA (V1V2)	EoE: *Neisseria* and *Corynebacterium*↑ non-EoE controls: *Streptococcus* and *Atopobium*↑ Reintroduce highly allergenic foods: *Ganulicatella* and *Campylobacter* genus↑
Benitez et al. (2021)	79 pediatric participants (33 active EoE, 36 inactive EoE, 10 non-EoE controls)	16 active EoE and 18 inactive EoE participants on TSS	esophageal biopsy samples	16S rRNA (V1V2)	EoE: *Alloprevotella*↓*Haemophilus*↑ EoE on TSS: *Haemophilus*↓*Candida*↑
Parashette et al. (2022)	41 pediatric participants (9 EoE, 4 PPIs-REE, 6 RE, 10 controls)	4 PPIs-REE and 6 EOE on PPIs, 1 EoE on TSS	esophageal biopsy samples	16S rRNA	*Rhodospirillales* was different between EoE and controls
Harris et al. (2015)	70 adult and pediatric participants (11 active EoE, 26 inactive EoE, 8 GERD, 25 controls)	5 active EoE, 12 inactive EoE participants and 11controls on PPIs, 12 inactive EoE participants on TSS	esophageal mucosa from the EST	16S rRNA (V1V2)	EoE: bacterial load ↑*Haemophilus*↑
Hiremath et al. (2019)	45 pediatric participants (15 active EoE, 11 inactive EoE, 19 non-EoE controls)	12 active EoE, 9 inactive EoE participants and 12 non-EoE controls on PPIs, 13 active EoE, 11 inactive EoE participants and 1 non-EoE controls on TSS	saliva samples	16S rRNA (V4)	active EoE: *Haemophilus*↑*Leptotrichiaceae*↓ non-EoE controls: *Neisseriaceae*↑ PPIs use: *Streptococcus*, *Corynebacterium*, and *Rothia*↑
Smith et al. (2015)	48 pediatric (6 active EoE, 7 treated EoE, 7 RE, 5 untreated IBD, 5 treated IBD, 18 controls)		cytology brush samples	16S rRNA (V4)	EoE: *Bacteroidetes*↑

EoE, Eosinophilic esophagitis; PPIs, proton-pump inhibitors; IBD, inflammatory bowel disease; TSS, topical swallowed steroids; PPIs-REE, proton pump inhibitors responsive esophageal eosinophilia; RE, reflux esophagitis; EST, esophageal string test; GERD, gastroesophageal reflux disease. ↑, increase; ↓, decrease.

**Table 2:** Summary of studies about the microbiota in adult EoE.

Study		Study population	Medication	Sample		Methods	Findings
Arias et al. (2018)		20 adults (10 EoE, 10 controls)		esophageal and duodenal biopsy samples		16S rRNA (V4)	Active EoE: bacterial load ↑ microbial load normalized following SFED
Facchin et al. (2022)		49 adults (29 EoE, 20 non-EoE controls)	26 EoE and 11controls on PPIs, 16 EoE on TSS	saliva samples(all participants), esophageal and gastric fundus biopsy samples (25 EoE and 5 controls)		16S rRNA (V3V4)	a microbial pattern of esophageal microbiota samples discriminate between active and inactive EoE
Ghisa et al. (2020)		16 adults (8 active EoE, 8 inactive EoE)		saliva samples, esophageal and gastric fundus biopsy samples		16S rRNA	active EoE: *Actinobacillus*, *Alloprevotella* and *Spirochaetes*↑ inactive EoE: *Firmicutes*↑
Johnson et al. (2021)		49 adults (24 EoE, 25 non-EoE controls)	24 EoE, 16 non-EoE controls on PPIs	esophageal biopsy samples		16S rRNA (V3V4)	no significant differences between EoE cases and non-EoE controls PPIs use: SR1, *Caulobacterales*, *Burkholderia*, *Eikenella* and *Kingella*↑
Laserna-Mendieta et al. (2021)		40 adults (30 active EoE, 10 non-EoE controls)	10 on PPIs, 10 on TSS	esophageal biopsy samples		16S rRNA (V4)	non-EoE controls: *Proteobacteria* ↑ *Bacteroidetes*↓ active EoE: *Parvimonas* and *Porphyromonas*↓ PPIs use: *Bacteroidetes* and *Fusobacteria*↓*Firmicutes*↑ TSS use: *Firmicutes*↓ *Proteobacteria*, *Bacteroidetes* and *Fusobacteria*↑
Norder Grusell et al. (2018)		27 adults (10 EoE, 17 GERD)		oral and esophageal biopsy samples		cultivation	Patients s with GERD had less bacterial diversity than EoE

EoE, eosinophilic esophagitis; PPIs, proton-pump inhibitors; TSS, topical swallowed steroids; SFED, six-food elimination diet; GERD. gastroesophageal reflux disease. ↑, increase; ↓, decrease.

In addition to the bacterial microbiota, other microorganisms may also be associated with EoE. Benitez et al. compared esophageal fungi in 69 children with EoE and 10 healthy controls. *Candida*, *Cladosporiaceae*, and *Malassezia* were the fungal taxa commonly present in esophageal samples across all groups. However, *Agaricomycetes*, *Candida*, *Cladosporiaceae*, and *Peniophora* were primarily present in samples from healthy controls (Benitez et al., 2021). Ghisa et al. found *Spirochetes* in esophageal samples from patients with EoE for the first time, which were more abundant in active patients than in inactive patients (Ghisa et al., 2020). However, these findings have not been validated in other studies.

The microbiota of the esophagus and oral cavity are correlated, and the oral microbiota of children with EoE is similar to that of the esophagus (Angerami Almeida et al., 2022). Hiremath et al. compared the salivary microbiota of 26 children with EoE and 19 controls and found a higher relative abundance of *Streptococcus* in children with active EoE than in healthy controls. *Haemophilus* was more abundant in the saliva of children with active EoE than in the saliva of children with inactive EoE and was positively related to the disease activity score (Hiremath et al., 2019). Another study compared the oral microbiota of 29 patients with EoE to that of 20 healthy controls and found that salivary flora distinguished patients with EoE from healthy controls (Facchin et al., 2022). These findings suggest that saliva sampling may be an effective and non-invasive microbial sampling method for the esophagus.

Apart from 16s rRNA gene sequencing, some studies have clarified the changes in the esophageal microbiota in patients with EoE by bacterial cultivation and found that the diversity of esophageal bacteria in patients with EoE was significantly higher than that in patients with gastroesophageal reflux disease, with *Alfa-streptococci* being the most abundant (Norder Grusell et al., 2018). However, current pediatric and adult esophageal microbiota studies rely on 16S gene sequencing, which has a limited sequencing depth. Traditional culture methods are limited to specific bacteria and may not facilitate comprehensive analysis. Moreover, some bacteria are difficult to identify in culture (Norder Grusell et al., 2018). Therefore, future studies should apply metagenomic sequencing combined with metabolomics to better describe the full genetic makeup and function of microbial communities in patients with EoE.

## 3 Potential microbial mechanisms involved in EoE

**Figure 1:**
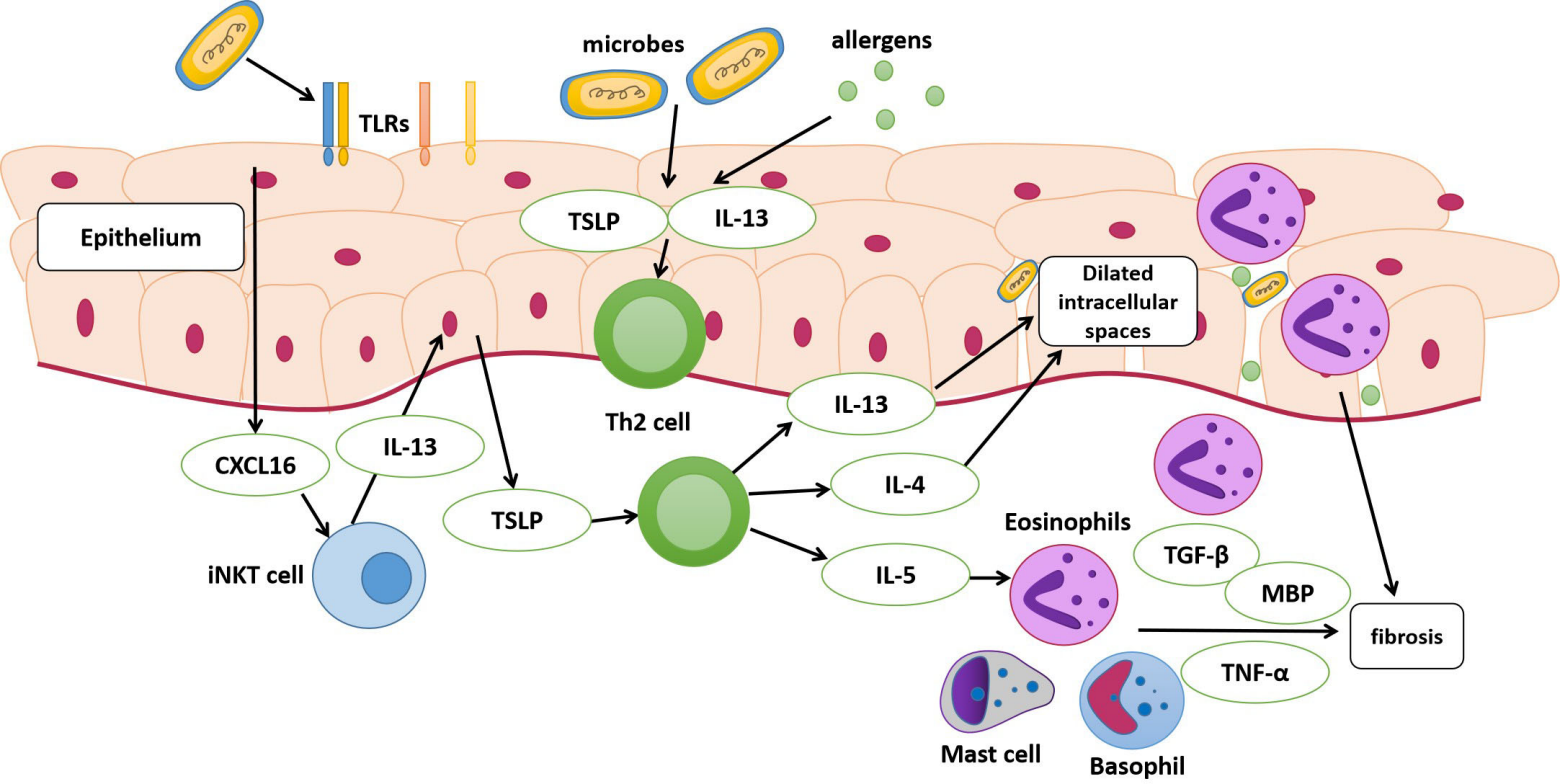
Pathophysiology of eosinophilic esophagitis (EoE). The dysbiosis of the esophageal microbiota may be a contributing factor to EoE. The esophageal epithelium of susceptible individuals with risk factors can release alarmin, IL-33, and thymic stromal lymphopoietin (TSLP) following the exposure to antigens and microbes. These cytokines stimulate the secretion of IL-13, IL-4, and IL-5 by T helper (Th2) cells. The activation of epithelial cells after exposure may modulate CXCL16 expression and then recruit invariant natural killer T cells (iNKT cell), which is regarded as a major source of Th2 cytokines and finally stimulate Th2 cells. IL-13 and IL-4 released by Th2 cells lead to dilated intracellular spaces, and disruption of esophageal mucosal barrier integrity eventually. IL-5 can stimulate the migration of eosinophils into the esophagus. Eosinophils, activated mast cells and basophils release cytokines, leading to the activation and proliferation of fibroblasts. MBP, major basic protein; TLRs, toll-like receptors.

An impaired epithelial barrier plays an important role in the development of EoE, through which allergens and microbes activate innate and adaptive immune cells with the subsequent production of chemokines and cytokines, leading to Th2 immune responses (Simon et al., 2016). The use of antibiotics and PPIs early in life may alter esophageal microbiota colonization (Jensen and Dellon, 2018). Alterations in commensal bacterial populations can elevate IgE production, increase basophil recruitment, and activate Th2 cell response (Hill et al., 2012). Toll-like receptors (TLRs) are pattern recognition receptors that distinguish pathogens from commensal bacterial populations (Jiménez-Dalmaroni et al., 2016). TLRs are mainly expressed in epithelial and lamina propria cells; the former are believed to be at the center of EoE pathogenesis (Rochman et al., 2018). A previous study found a high esophageal bacterial load and overexpression of TLRs in patients with EoE, suggesting that the microbiota and its products may activate TLRs and increase the release of inflammatory factors from esophageal epithelial cells, resulting in the progression of EoE (Arias et al., 2018). Moreover, in early EoE, interactions between epithelial cells and esophageal microbiota may modulate CXCL16 expression and recruit invariant natural killer T cells, a major source of Th2 cytokines, to the esophageal epithelium (Arias and Lucendo, 2019). These findings suggest that dysbiosis of the esophageal microbiota may contribute to EoE (**Figure 1**). However, several studies have demonstrated dysbiosis of the esophageal microbiota as a consequence of EoE. Masterson et al. indicated that the expression of hypoxia-inducible factor-1α is inhibited in patients with active EoE, followed by the selective downregulation of the tight junction protein CLDN1, resulting in esophageal barrier dysfunction (Masterson et al., 2019). In addition, a decrease in hypoxia-inducible factor-1α also leads to decreased secretion of the antimicrobial peptide β-defensin by the epithelium (Kelly et al., 2013), finally resulting in an increased bacterial load (Angerami Almeida et al., 2022). Eosinophils that infiltrate the esophagus also secrete defensins that affect the local microbiome (Kanikowska et al., 2021).

The role of *Helicobacter pylori* in EoE remains controversial. Some studies have found that *H. pylori* promotes the expression of IFN-γ and IL-17, resulting in the proliferation of Th1 and Th17 cells, consequent downregulation of Th2 cells, and inhibition of the immune response, thus playing a protective role in EoE (Dowling et al., 2019). A series of observational studies have also shown an inverse association between *H. pylori* infection rates and EoE prevalence (Furuta et al., 2013; Doulberis et al., 2020). However, a recent large case-control study did not find evidence of a protective effect of *H. pylori* against EoE (Molina-Infante et al., 2018), suggesting that the relationship between reduced *H. pylori* infection and increased EoE prevalence is complex and noncausal (Talley and Walker, 2018).

## 4 Role of microbiota in EoE treatment

Dietary modifications and pharmacological drugs (including PPIs and corticosteroids) are currently the primary treatment options for EoE (Gonsalves and Aceves, 2020). Dietary modifications and pharmacological drugs can modulate the esophageal microbiota (**Table 3**), suggesting that the microbiota may be an important biomarker for predicting and evaluating therapeutic effects on EoE. Microbial interventions can also be used to manage EoE.

**Table 3:** Effect of Pharmacological Drugs on Esophageal Microbiota.

Study		Study population	Medication	Sample	Findings	
Laserna-Mendieta et al. (2021)		10 adults with active EoE	10 patients on PPIs use (8 weeks, double-dose)	esophageal biopsy samples	compared with baseline: *Bacteroidetes* and *Fusobacteria* ↓*Firmicutes* ↑	
Hiremath et al. (2019)		26 pediatric participants (15 active EoE, 11 inactive EoE)	21 patients on PPIs use; 5 patients with no PPIs use	saliva samples	compared with no PPIs use: PPIs use was not significantly associated with any microbiome changes	
Harris et al. (2015)		37 adult and pediatric participants (11 active EoE, 26 inactive EoE)	17 patients on PPIs use; 20 patients with no PPIs use	esophageal mucosa from the EST	compared with no PPIs use: PPIs use was not significantly associated with any microbiome changes	
Laserna-Mendieta et al. (2021)		10 adults with active EoE	10 patients on TSS (12 weeks)	esophageal biopsy samples	compared with baseline: *Firmicutes*↓ *Proteobacteria*, *Bacteroidetes* and *Fusobacteria*↑
Benitez et al. (2021)		69 pediatric participants (33 active EoE, 36 inactive EoE)	34 patients on TSS; 35 with no TSS (9 patients receive TSS later)	esophageal biopsy samples	compared with no TSS use: *Actinobacillus* and *Haemophilus*↓*Candida*↑ compared with baseline: *Actinobacillus*↓
Hiremath et al. (2019)		26 pediatric participants (15 active EoE, 11 inactive EoE)	8 patients on TSS for EoE; 16 patients use steroids for other disease; 2 patients with no steroids	saliva samples	compared with no TSS use: TSS use was not significantly associated with any microbiome changes	

EoE, eosinophilic esophagitis; PPIs, proton-pump inhibitors; TSS, topical swallowed steroids; EST, esophageal string test. ↑, increase; ↓, decrease.

### 4.1 Dietary modification

The targeted elimination diet, six-food elimination diet (SFED), and elemental diet are currently used to treat EoE and have proven clinical efficacy. Kelly et al. demonstrated the therapeutic effect of dietary modification on EoE for the first time (Kelly et al., 1995). A retrospective study analyzed 31 adult patients with EoE who received a targeted elimination diet or SFED and found that symptoms and endoscopic appearance improved in 71% and 54% of patients, respectively (Wolf et al., 2014). It was found that the increased bacterial load in the esophagus normalized after SFED in adult patients with EoE (Arias et al., 2018). However, another study demonstrated that esophageal bacterial load in patients with EoE was not affected by dietary regulation (Harris et al., 2015). Benitez et al. found no significant differences in the esophageal microbiota after removing food(s) from SFED in patients with active EoE. However, the *Granulicatella* and *Campylobacter* genera were enriched in the esophageal microenvironment following the addition of highly allergenic foods to the diet of patients with inactive EoE (Benitez et al., 2015). A recent study demonstrated that α diversity decreased among patients with EoE who underwent a food elimination diet, and the abundance of *Firmicutes* slightly increased (Laserna-Mendieta et al., 2021).

### 4.2 Pharmacological drugs

#### 4.2.1 Proton pump inhibitors

In recent years, with the proven clinical efficacy of PPIs the definition of EoE has been updated to include PPIs-responsive esophageal eosinophilia, in which symptoms and histology improve after PPIs treatment (Dellon et al., 2018). PPIs are widely accepted as first-line medications for EoE, with a 30% – 70% response rate (Gonsalves and Aceves, 2020). A multicenter observational study showed a 49% histological response rate and a 71% clinical response rate to PPIs treatment (Laserna-Mendieta et al., 2020). PPIs can affect the esophageal microbiota by altering the pH and microenvironment (Vesper et al., 2009). One study found that the abundance of *Bacteroidetes* and *Fusobacteria* decreased slightly from baseline after receiving PPIs, whereas that of *Firmicutes* increased in patients with EoE(Laserna-Mendieta et al., 2021). PPIs use was associated with a high abundance of *Streptococcus*, *Corynebacterium*, and *Rothia*. However, no evidence of PPIs-induced microbiome changes was found in children with EoE (Hiremath et al., 2019). Another study observed an increased abundance of *Proteobacteria* and *Aggregatibacter* and a decreased abundance of *Streptococcus* in patients with gastroesophageal reflux disease treated with PPIs but not in patients with EoE (Harris et al., 2015).

#### 4.2.2 Hormone therapy

Topical swallowed steroids, such as fluticasone propionate or budesonide, are currently among the most effective first-line therapies for children and adults with EoE. Corticosteroids inhibit IL-13 transcription *in vivo*, reduce eosinophilic and T cell infiltration, downregulate mast cell-related genes, decrease fibrosis, and restore esophageal motility (Racca et al., 2021). A previous study comparing the efficacies of topical and systemic corticosteroids demonstrated histological and clinical remission with both treatment methods. Systemic administration showed greater histological improvement than topical administration but no significant advantage in symptom resolution, relapse rates, or time to relapse (Schaefer et al., 2008). However, given the adverse effects, systemic administration is only used in patients with critical EoE and symptoms of severe dysphagia or significant weight loss (Dellon et al., 2013). One study found that corticosteroids did not change the diversity or microbiota abundance in children with EoE (Hiremath et al., 2019). Benitez et al. indicated that the relative abundance of *Actinobacillus* was lower in children treated with corticosteroids than in steroid-naïve populations. The relative abundance of *Haemophilus* was lower in active steroid non-responders than in children with active steroid-naïve EoE. The abundance of *Candida* significantly increased in children with inactive EoE treated with corticosteroids compared to that in untreated inactive EoE subjects, whereas *Cladosporiaceae* significantly increased in inactive steroid responders. Changes in esophageal microbiota were assessed in the same patient before and after corticosteroid therapy. The abundance of *Actinobacillus* decreased, which was consistent with a cross-sectional comparison, whereas there was no significant change in the relative abundance of *Haemophilus* or fungi (Benitez et al., 2021). Another study demonstrated that patients treated with corticosteroids had a decreased abundance of *Firmicutes*, a relatively high abundance of *Proteobacteria*, *Bacteroidetes*, and *Fusobacteria*, and a microbiota composition similar to that of controls. In addition, analysis of the predicted function indicated that the metabolism of sulphur groups, 4-aminobutanoate, ornithine/arginine, and cell peptidoglycan can be affected by microbial changes related to the treatment received. These alterations may be involved in the pathogenesis of EoE (Laserna-Mendieta et al., 2021).

#### 4.2.3 Microbiota interventions

Microbiota modulators, including probiotics and prebiotics, can reshape or adjust microbiota composition to improve disease. The efficacy of probiotics has been demonstrated in various diseases related to microbiota dysbiosis, including antibiotic-associated diarrhea, inflammatory bowel disease, and irritable bowel syndrome (Kim et al., 2019). Potential mechanisms include inhibition of pathogenic bacterial adhesion, enhanced mucosal barrier function, modulation of innate and adaptive immune systems, secretion of bioactive metabolites, and regulation of the enteric and central nervous systems (Liu et al., 2018). The effectiveness of probiotics for the treatment of EoE has been explored in animal studies. In a mouse model of EoE, supplementation with *Lactococcus lactis* inhibited IL-5 production and reduced esophageal eosinophilic infiltration (Holvoet et al., 2013; Holvoet et al., 2016). In addition, *Clostridia* regulate intrinsic lymphocyte function and epithelial permeability to prevent allergen sensitization (Stefka et al., 2014), and the abundance of *Clostridia* is significantly reduced in the gut of patients with EoE (Kashyap et al., 2019), suggesting that it can be used as a potential intervention target. Although prebiotics can promote the growth of beneficial bacteria, there is currently a lack of research on their efficacy in treating EoE. Furthermore, fecal microbiota transplantation is used to treat several diseases as it restores the microbiota (Ooijevaar et al., 2019). It has been reported that fecal microbiota transplantation can restore the esophageal microbiota (Brusilovsky et al., 2022). Fecal microbiota transplantation may have therapeutic potential in EoE and corresponding preclinical and clinical studies could be instrumental in furthering treatment.

## 5 Discussion

In this review, we summarize the published EoE-related esophageal microbiota studies. The main findings showed that the esophageal microbiome was altered and was involved in the pathophysiology of EoE. The microbiome may serve as a potential therapeutic target. However, there are differences among the conclusions of various studies. Further research is required to investigate microbiota composition and role in EoE.

Currently, 16S rRNA sequencing is the most widely used sequencing method, providing an efficient and economical way to determine microbial community structure and composition. However, this method has the following drawbacks. First, there is no consensus on which variable region is the most suitable for microbiota identification in patients with EoE, and the difference leads to identification bias based on primers selection (Busing et al., 2021). There were significant differences in the selection of primers in the studies included in this review, which may be one of the reasons for the differences between study conclusions. Secondly, the species resolution achieved with 16S rRNA sequencing was lower than that achieved with metagenomic sequencing. The former usually cannot accurately identify the species or distinguish similar strains, leading to bias in microbiota identification. This method can only identify a microbial database based on short sequences (Angerami Almeida et al., 2022). Third, 16S rRNA sequencing cannot distinguish dead bacteria, which interfere with results (Norder Grusell et al., 2018). Finally, 16S rRNA sequencing tends to ignore information on fungi and viruses, resulting in limited conclusions. Metagenomics is better than 16S rRNA in terms of sequencing depth, which can compensate for some of the limitations of 16S rRNA (Angerami Almeida et al., 2022). However, metagenomic sequencing has not been used to identify the esophageal microbiota of patients with EoE. Moreover, little work has been conducted in metabolomics and metaproteomics, which may provide clues for identifying potential therapeutic targets and pathogenesis. Therefore, future studies should test metagenomic sequencing combined with metagenomics and metabolomics in the assessment of the genetic makeup and function of microbial communities.

However, different sampling sites may yield conflicting results. Enrichment of *Neisseria* was observed in esophageal biopsy samples from patients with EoE (Benitez et al., 2015), whereas an increased relative abundance of *Neisseria* was found in saliva samples taken from non-EoE controls (Hiremath et al., 2019). Several studies that used esophageal samples showed a decreased abundance of *Bacteroidetes* in patients with EoE (Smith et al., 2015; Benitez et al., 2021), whereas Kashyap et al. observed a significant increase in fecal samples of patients with EoE (Kashyap et al., 2019). Although saliva sampling is expected to be an effective and noninvasive microbial sampling method for the esophagus, esophageal biopsy remains the gold standard, and the relationship between the two needs to be further explored.

Studies have shown that all current first-line EoE therapies can affect the esophageal microbiota; however, definitive conclusions about the alterations of microbes after treatment cannot be drawn. This may be related to the small sample sizes and the short time between the last sampling time point and the end of treatment. Changes in the esophageal microbiota are expected to provide a new target for EoE treatment.

## 6 Conclusions

The esophageal microbiota is involved in the development and occurrence of EoE. Research on fluctuations in the esophageal microbiota and their effects on patients with EoE could help identify new therapeutic targets and improve patient prognosis. The esophageal microbiome is altered in patients with EoE compared to that in healthy controls, characterized by an increase in the abundance of *Haemophilus* and a decrease in that of *Firmicutes*. Moreover, EoE therapies can modulate the esophageal microbiota, and microbiota modulators are potential ways to cure EoE. However, studies on EoE-related esophageal microbiota are limited. Additionally, certain conclusions have not been validated by other studies, or conflicting results have been obtained. This may be due to small sample sizes or the interference of medications. Sequencing methods are also important in this regard. 16S rRNA sequencing has limited sequencing depth and has been used in most studies. Future studies should apply metagenomic sequencing to better describe full genetic makeup. In addition, there remain gaps in multi-omics studies. Little work has been done on metabolomics and metaproteomics, which may provide information on the function of a microbial community and give insight into potential biomarkers for noninvasive disease monitoring and therapeutic outcomes. In conclusion, the clinical value of esophageal microbiota in EoE remains to be explored.

## Author contributions

XZ and NZ: Writing - Original Draft; ZW: Conceptualization, Writing - Review and Editing. All authors contributed to the article and approved the submitted version.
